# Fostering “Education”: Do Extracellular Vesicles Exploit Their Own Delivery Code?

**DOI:** 10.3390/cells10071741

**Published:** 2021-07-09

**Authors:** Mayra Paolillo, Sergio Comincini, Sergio Schinelli

**Affiliations:** 1 Department of Drug Sciences, University of Pavia, 27100 Pavia, Italy; sergio.schinelli@unipv.it; 2 Department of Biology and Biotechnology, University of Pavia, 27100 Pavia, Italy; sergio.comincini@unipv.it

**Keywords:** extracellular vesicles, exosomes, click chemistry, recipient cells, glyco-proteomics

## Abstract

Extracellular vesicles (EVs), comprising large microvesicles (MVs) and exosomes (EXs), play a key role in intercellular communication, both in physiological and in a wide variety of pathological conditions. However, the education of EV target cells has so far mainly been investigated as a function of EX cargo, while few studies have focused on the characterization of EV surface membrane molecules and the mechanisms that mediate the addressability of specific EVs to different cell types and tissues. Identifying these mechanisms will help fulfill the diagnostic, prognostic, and therapeutic promises fueled by our growing knowledge of EVs. In this review, we first discuss published studies on the presumed EV “delivery code” and on the combinations of the hypothesized EV surface membrane “sender” and “recipient” molecules that may mediate EV targeting in intercellular communication. Then we briefly review the main experimental approaches and techniques, and the bioinformatic tools that can be used to identify and characterize the structure and functional role of EV surface membrane molecules. In the final part, we present innovative techniques and directions for future research that would improve and deepen our understandings of EV-cell targeting.

## 1. Introduction

Intensified research into extracellular vesicles (EVs) in the last 20 years has revealed a new paradigm in intercellular communication. Originally regarded as mere garbage packaging, EVs rose to the privileged status of universal mechanism used by any cell type to exchange almost any molecule involved in cell physiology and pathology [1,2,3]. The basic principle underlying EV-dependent communication is quite simple, and resembles that of most hormones: EVs are first released by their donor cells into the extracellular space and then taken up by other close or distant recipient cells. The main purpose of this efficient communication system is the education of recipient cells by donor cells; to carry out this process, EVs exploit a variety of biological molecules (such as DNA, RNA, lipids, proteins and enzymes) packed as cargo. 

The field of EVs has attracted a lot of interest from biomedical scientists in the last 10 years, leading to thousands of published experimental papers and reviews, covering almost every area of cell biology, physiology, and pathology. Although we understand in the literature alternative and more detailed EVs classifications have been proposed, for purpose of simplicity in this review we will distinguish two broad types of EVs based mainly on their mechanism of formation and their size: microvesicles (MVs) and exosomes (EXs). 

Readers interested in a detailed account of EVs features, such as EVs release mechanisms, role, composition, and functional effects elicited by EV cargo, could refer to several of the comprehensive reviews that have appeared recently in the literature [4,5,6,7,8]. 

An important but seldom-addressed issue in this mass of often overlapping information is: do EVs use a sort of “delivery code”? EVs must indeed maximize a key step to unleash the potential educational effects of their molecular cargo: uptake by recipient cells. In simple words, such a delivery code would have to include two separate subtypes: a donor delivery code and a recipient delivery code. This hypothesis raises several intriguing questions: (a) is it possible to deduce the type of parent donor cells and the type of recipient cells by analyzing the molecules exposed on the outer membrane of EVs?; (b) do the donor and recipient delivery codes leverage their own different patterns of “sender” and “recipient” molecules to perform their duty? Cracking this presumed EV delivery code by leveraging the data from studies aimed at deciphering the identity and functional role of “sender” and “recipient” molecules, would have profound implications for a wide range of crucial topics in cell biology. A variety of mechanisms is deployed in the formation and release of EV subtypes: small EVs such as EXs derive from endosomal compartments, while larger EVs are assembled via invagination of cell membranes, suggesting that EVs protein expression patterns should resemble those of their parent cells. The identification of biomarkers has practical applications both in EV classification and in the search for reliable indicators for diagnosis and drug monitoring in several pathologies. The quest to find these indicators is especially relevant to cancer and metastasis studies, since the identification of EVs surface membrane molecules, especially glycoproteins, proteins, and lipids, would enable the inference of several features of their cancer parental cells [9]. Indeed, many studies have clearly shown that cancer cell-derived EXs can be separated in various ways [10] from the plasma of patients affected by diverse cancer types. Multi-omics analysis of these cancer cell-derived EVs would allow the investigation, via liquid biopsies, of key parameters involved in the response of parental cancer cells to therapy and in the adaptive progression of the metastatic process. 

One of the most promising fields of application, derived from deeper insights into the ability of EVs to target specific tissue or cells, deals with EVs as a potential tool for drug delivery [11,12]. EVs display important features and advantages over conventional drug delivery systems, (a) EVs are not immunogenic; (b) EVs can be loaded with drugs and other therapeutic agents; (c) EVs can cross almost any biological barrier; (d) EV surface membrane molecules can be engineered to modulate their uptake into recipient cells; and finally (e) EVs are highly suitable as a delivery tool for gene therapy or in small RNA-mediated interference applications. However, several important problems need to be addressed before these approaches can be approved by drug approval agencies. 

The main problems are the homogeneous isolation and standardized large-scale production of EVs necessary to implement drug loading systems. EVs display a variable composition that depends upon several parameters such as parental cell type and cellular activation state, conditions of in vitro propagation and growth biogenesis pathway, which is in turn affected by intracellular cargo sorting routes [13,14]. 

Methods for loading EVs or modified EVs with drugs suitable for encapsulation [15] are being investigated but this strategy needs refining due to its low efficiency and the risk of toxicity. 

Finally, the feature that most qualifies EVs as a promising vehicle for drug delivery is the potential to modify and engineer EV surface membrane molecules to enhance their selectivity and specificity toward target cells [16]. This feature has been fruitfully exploited in harnessing EXs as a precise and reliable system for delivering drugs to treat the brain: the bioavailability of such drugs is severely impaired by the blood brain barrier (BBB) [17].

Recent years have seen a rapid increase in our knowledge of the many features shared by viruses and EVs, such as size, packaging mechanisms and patterns of release [18]. Notably, the main difference between viruses and EVs is the content of their molecular cargo and the fact that EVs are not infectious, a key advantage for future applications (see the conclusion section). Perhaps the most important common functional property is that both viruses and EVs rely on the glycosylation of their surface membrane or envelope proteins to bind with specific surface membrane components of target cells [19]. This notion has been validated by a novel finding that highlights the crucial role of glycans in the interaction between virus glycoproteins and their targeted receptors, implicated in the mechanism of accession to cells. The authors conducted a large-scale bioinformatics analysis to identify specific glycan-attached amino acids in the SARS-CoV-2 spike protein, which form the 3D conformations of the spike receptor binding domain that interacts with the cell surface membrane ACE-2 receptor [20]. Although this virtual result requires confirmation by different and complementary experimental techniques, it could represent a starting point for studies aimed at investigating the composition, similarities, and difference between EVs and virus glycoproteins. Future perspectives and possibilities raised by this issue are discussed in the future trends section at the end of the review. 

In this review, we will first illustrate the principal techniques used to analyze EV surface membrane molecules and then give an overview of experimental works that have attempted to deduce their functional effects. We conclude by suggesting future trends and innovative approaches to improve current knowledge of EV-recipient cell interactions. 

## 2. EV Analysis

The initial step in the analysis of proteomic patterns relies on the methods used to isolate EVs from different sources such as human biological fluids, tissues, and cell cultures. Several excellent reviews [21,22,23] have described in detail all the critical parameters, such as time, purity, complexity, and yield of the most-used isolation and purification methods, thus providing a guide to the most suitable and feasible procedures as a function of downstream applications. Here we will briefly discuss the most-used separation methods, bearing clearly in mind that these methods, with few exceptions, have been devised mainly to purify only exosomes (EXs), and their performance in separating MVs is not known.

The most common method is based on a three-step ultracentrifugation which allows separation of MVs from EXs. However, this protocol suffers from low specificity, showing variable overlap between MVs and EXs when their size is measured by NTA assay. A refined version of this method is a density gradient UC that can separate MVs by density with extreme precision and reliability, but on the downside this experimental protocol is rather tedious and time-consuming. 

To avoid the formation of tight clusters of MVs, some companies have developed kits based on the capability of certain polymers, such as PEG or dextran, to trap and then precipitate MVs at low speed. These applications are less harsh than UC; however, they have major pitfalls: they lack specificity and selectivity toward MV subpopulations, and they necessitate an additional clean-up test to remove salts and other impurities before downstream analyses. Other less-employed methods used for EV separation include ultrafiltration, size-exclusion chromatography (SEC) and immunoaffinity capture (IC) alone or coupled with FACS-assisted separation. Although IC relies on the use of beads or magnetic particles, FACS can sort and analyze subpopulations of EVs by leveraging fluorescent labeled antibodies directed against EV surface membrane epitopes. The procedure is very gentle and displays an extremely high-throughput yield, but it has three main drawbacks: highly specific antibodies with strong affinity must be employed; EVs lacking specific antigens on their surface are lost; and it requires dedicated and expensive nano-FACS equipment with submicron separation capability, operated by skilled personnel. 

In addition, none of the isolation methods separates EVs with a non-homogeneous degree of purity, meaning the isolate may contain contaminant non-EV-like particles, together with other, soluble contaminants [24,25]. Unfortunately, most EV isolation method protocols do not specify whether the procedure retains the integrity and functionality of surface membrane molecules, and future studies are required to carefully address this important issue. 

An innovative, efficient EX isolation and detection system called EXODUS allows label-free separation of exosomes from different biofluids [26]. This approach, based on negative pressure oscillation and double coupled harmonic oscillator-enabled membrane vibration, displays several improvements over existing classical EX isolation protocols in terms of speed, yield, and purity, thus allowing high resolution and high-throughput EX analysis. 

The methods employed to characterize EV surface membrane proteins isolated from donor tissue, liquid, or cells, can be divided in two main categories: (a) the direct approach based on the plain MS analysis of all proteins extracted, followed by the use of bioinformatics algorithms for the assignment of membrane topology, (b) indirect approaches based on the treatment of EVs with tryptic enzymes or chemical reagents that cleave only molecules protruding from the membrane surface, followed by MS analysis of the released fragments. 

The direct proteomic approach analyzes total EV lysate partially enriched by preparatory separation. Usually performed with LC, separation is followed by analytical identification of fragmented protein by tandem MS. This approach has been instrumental in enabling the assembly of EV atlases or databases describing hundreds or thousands of EV proteins [27,28,29,30,31,32]. Unfortunately, this methodology is not conclusive in pinpointing EV surface membrane proteins because it does not discriminate between cargo proteins and the inside or outside orientation of putative surface membrane proteins. A partial solution for this impasse is represented by bioinformatics computational algorithms, listed in Table 1, that can discriminate membrane spanning proteins among the whole group of proteins detected by MS [33,34]. However, although this resource is quite powerful and reliable, the inside-out or outside-in orientation of surface membrane proteins remains challenging to determine from MS data, and is sometimes ambiguous even for the best algorithms. This critical drawback requires additional information that must be obtained with a complementary experimental approach able to identify outside-protruding epitopes located on EV membranes with more precision. 

In indirect approaches, selective fragments or chemically reactive groups located on the EV surface membrane, react with specific agents as a preliminary step towards subsequent isolation and less cumbersome and more targeted MS analysis. Indirect approaches themselves can basically be divided into two main subtypes: the shaving procedure and the chemical modification of primary amines.

### 2.1. Shaving

The surface shaving method is based on the treatment of intact prokaryotic or eukaryotic cells with proteolytic enzymes followed by the isolation of cleaved peptides that are usually identified by MS [44]. The main purpose of this technique is the experimental deduction of the portions or peptide fragments of proteins that span the membranes and protrude from the outside. This procedure has been extensively applied to bacterial cells and to various eukaryotic organism [45,46], but its implementation in the EV field is lagging behind. Under appropriate and controlled conditions, the shaving treatment does not substantially change the size and other physical features of treated EVs but, interestingly, reduces their mobility thus underscoring once again the functional role of surface membrane epitopes in regulating EV interactions [47]. 

The topology of EV surface membrane proteins was investigated by means of a proteomic approach based on proteinase treatment combined with biotin tagging [48]. Quite surprisingly, FACS analysis and fluorescence imaging with antibody-recognizing cytoplasmatic epitopes revealed that several EV membrane proteins are embedded in the EV membrane in a topologically reverse orientation. This result clearly stresses the need to combine experimental and theoretical approaches to disclose the relevance of surface membrane protein orientations in EV functionality. However, such an approach would have to be carefully planned to avoid misinterpretation of results due to (a) possible inaccessibility of proteolytic enzymes to protein epitopes, or pockets masked by glycans; and (b) wrong shaving procedure conditions that might lead to the formation of holes in the membrane that would allow proteolytic enzymes to also cleave intracellular or inner surface membrane proteins. Another cause of failure of this procedure might be a limited quantity of cleaved EV proteins, resulting in very small amounts of peptide fragments that could go undetected by subsequent MS analysis. Despite these drawbacks, it is worth conducting future studies to standardize shaving experimental conditions to provide a complementary method for the validation of bioinformatic analysis carried out on whole EV protein lysate.

### 2.2. Primary Amine Reactions 

The main goal of chemical modification of surface membrane molecules is the insertion of specific chemical groups that could serve as docking or acceptor sites to be used as separating tools. One of the most commonly used systems is protein biotinylation via coupling of primary amine residues to NHS-biotin; the protein-NHS-biotin complex is then separated from other interfering proteins by affinity chromatography with avidin or streptavidin beads. Two main types of biotin coupling reagents are available: cell-permeable sulfo-NHS-biotin labels the proteins in the cytosol or on the inner side of the EV membrane while the cell-impermeable sulfo-NHS-LC-LC-Biotin labels the proteins on the outer side of intact cells or EV membranes. This latter reagent, due to a larger spacer arm between the biotin and amine reactive linker, could reduce the negative effect of steric hindrances thus improving labeling efficiency at crowded cell or EV surfaces. Because amine residues are sometimes not readily available for coupling, their expression could be enhanced by selective treatment of EV membranes with simple molecules, as in the elegant procedure proposed recently by Sharpless’s group [49]. To reduce problems associated with the use of copper-catalyzed azide–alkyne cycloaddition (CuAAC) in click chemistry reactions, the authors treated primary amines with fluoro-sulfuryl azide (FSO_2_N_3_), leading to the formation of azide residues. Although the applicability of this method to the EV field awaits further confirmation, it holds great promise for expanding the arsenal of chemical modifications of surface membrane primary amine-bearing molecules.

## 3. EV Targeting

The uptake of EVs by recipient cells mainly occurs through membrane fusion and endocytosis [14,50], which require the participation of distinct classes of molecules. The most abundant and best-characterized ligands implicated in EV-cell interactions are glycoproteins, lipids, and glycans, but the role of other surface membrane molecules such lectins, heparan sulfates proteoglycans and EGFR should not be dismissed [51].

To have some glimpses of the molecules located on outer surface membranes that could mediate in the EV-cell interactions [52], it is useful to look at reports describing the selective and specific uptake of certain EV subpopulations by target tissues or cells [53]. Functional studies conducted so far have used one of two protocols: the former investigates the uptake of one EV population by distinct types of recipient cells, while the latter is based on a sort of reverse approach in which several EVs derived from different donor cell are tested for their intrinsic ability to internalize into one specific cell type. In addition, EV interaction with recipient cells depends on several parameters that include, but are not limited to, transformation with ectopic expression of oncogenes [54], activation of certain signaling pathways and different proliferation and mitotic states. For example, EXs from oligodendroglia precursors are preferentially internalized by microglia but not by other brain cell types [55], and bone marrow dendritic cell (DC) EXs are internalized by splenic conventional DCs rather than by other immune cells [56]. In certain cases, binding and internalization mechanisms may differ between cell types; the kinetics and modality of uptake of microglia-derived MVs are different for microglia and astrocytes [57] and the same has been observed for EXs derived from leukemic cells toward recipient phagocytic cells [58]. Several in vivo experiments have confirmed targeted localization of EVs derived from distinct cell types toward diverse organs and tissues. Human embryonic kidney cell-derived EVs injected into mice localize mainly to the liver and the spleen [59,60] and mesenchymal stem cell-derived EVs accumulated preferentially in the organs where they can modulate injury recovery. Notably, melanoma-derived EXs may enhance metastatic processes in lungs, bone, liver and spleen, probably educating recipient cells in these compartments and thus preparing the soil for the formation of the pre-metastatic tumoral niche [61]. 

In the central nervous system, EVs have been found to play a crucial role in intercellular communication in both physiological and pathological conditions. For example, in brain tumors, astrocyte-derived EVs are involved in reciprocal crosstalk with glioblastoma (GBM) cells [62] which may mediate pro- or antitumor roles of astrocytes in tumor development. Another study broadened the functional role of astrocytes showing that upon activation by interleukins, these cells could affect neuronal uptake, differentiation, and firing via enrichment of surface membrane proteins such as integrins and the major histocompatibility complex [63]. That EVs have a differential affinity for specific target cells is supported by the finding that neuroblastoma-derived EXs interact with hippocampal neurons and glial cells but are preferentially taken up by glial cells whereas cortical neuron-derived EXs are endocytosed almost exclusively by hippocampal neurons [64]. This report suggests that at least in the central nervous system, EV targeting may entail both specific and non-specific mechanisms, probably reflecting the selective pattern of surface membrane proteins. However, although these studies indicate a promising direction for future in vivo and in vitro models, it should be remarked that few of them have clarified the type and composition of surface membrane molecules involved in EV-cell interactions. This consideration urges a widening of focus in future investigation of EV-cell interactions from the cellular level to encompass the structural molecular level. 

Quite surprisingly, only two studies have investigated the molecular identity of conjectured sender or receiver biomolecules expressed on the outer membrane surface. In the first seminal report [65], the authors found that EXs derived from different tumor cell lines display organotropism toward specific tissues to prepare the pre-metastatic niche. Proteomic analysis demonstrated that this mechanism is mainly mediated by subclasses of EX integrins: specifically, integrins α6β4 and α6β1 preferentially trigger lung metastasis, while integrin αvβ5 preferentially triggers liver metastasis. In addition, functional experiments have shown that integrin engagement with recipient cells activates the Src-dependent signaling pathway and induces S100 gene expression. These data strongly suggest that the characterization of tumor-derived EX integrin patterns represents a very informative parameter to predict organ-specific metastasis. 

The pivotal role exerted by cellular integrins [66,67] and integrin expression in EVs in cancer-dependent mechanisms [68,69] has been further confirmed by a study which showed that in mice, blood or cancer-derived EVs modulate anchorage-independent growth of prostate cancer by recruiting β1 integrins [70]. In another study [71], heparan sulfate proteoglycans (HSPGs), localized on the recipient cells’ outer membrane surface, acted as receptors for cancer cell-derived EXs. This result could have a critical impact on EV biology, because it strongly suggests that the pharmacological perturbation of the HSPG-dependent uptake route, together with identification of the EV binding partners of HSPG, could be a potential target for the modulation of EX-mediated tumor development. However, the results of these two studies should be taken with caution: those of the first report have not been fully reproduced and therefore require confirmation; while in the second study, the overexpression of HSPG in target cells may invalidate the conclusion, likewise necessitating validation in similar but more physiological experimental conditions.

### 3.1. EV Biomarkers

EV biomarkers may be the key to classifying EVs subtypes in addition to being potentially reliable cancer biomarkers [72] that could improve the predictive performance of liquid biopsies in clinical oncology. In support of this assumption, extensive profiling of the pancreatic ductal adenocarcinoma (PDAC) EX surfaceome revealed multiple PDAC-specific biomarker candidates [73]. In a similar report [74], in murine mast cell and human urine-derived EXs, tissue-specific common protein patterns involved in transport, signaling, and cytoskeletal proteins were detected. Proteomic analysis of EVs derived from human dendritic cells [75] has allowed the classification of EVs based on the expression pattern of five protein categories, thus allowing the authors to implement an EV immuno-separation method based on the use of the tetraspanins CD63, CD81, or CD9 as associated markers. In agreement with this finding, a recent MS-based proteomic approach defined markers in human plasma EVs that can distinguish between individuals with or without certain cancer types [76]. 

A targeted label-free proteomic strategy (SWATH-MS) demonstrated that certain common EV markers (CD9, CD63, ALIX, TSG101 and HSP70) were enriched in urinary EXs as compared to MVs and urinary free proteins [77], validating this approach for distinguishing between MV and EX protein expression patterns. In another application of proteomic analysis of EVs derived from human colon and lung cancer cell lines [78], the authors found that among nearly 30 cell line-specific markers, several proteins were involved in integrin-, Rap1-, and EGFR-dependent signaling pathways, further strengthening the idea that EV biomarkers may identify their parental tumor cells.

### 3.2. EV Engineering

Several chemical engineering approaches have been proposed to enhance the ability of EVs to be taken up by recipient cells [79,80,81,82]. These modifications are mainly aimed at improving the selectivity of drug cargo delivery to reduce the adverse effects of drugs; and at synthesizing novel imaging reagents that will enable precise and early diagnosis of illnesses. The addition of the Arg-Gly-Asp (RGD) peptide on the EXs outer membrane surface improves targeting of blood vessels [17] thus enhancing not only the efficacy of therapeutic intervention against angiogenesis but also the performance of imaging procedures via click chemistry-dependent metabolic labeling. In another quite similar approach [83], the conjugation of the c(RGDyK) peptide to EX surface membrane proteins, via bio-orthogonal click chemistry, increased cRGDyK-EXs targeting in an in vivo mouse model of brain ischemia. More strikingly, when the same cRGDyK-EXs were preloaded with the drug curcumin, suppression of inflammation was observed, giving credit to the promises of EXs as a drug delivery tool.

## 4. Glycoproteomics—A Code within a Code?

Glycosylation is one of the most common post-translation modifications (PTM) of proteins and the most relevant in terms of its impact on several key cellular events such as cell-cell interaction, cell dissemination, cell attachment and immune response toward host pathogens [84,85]. These mechanisms are mainly mediated by a complex and dynamic re-arrangement of a wide variety of glycans bound to and protruding from proteins present on the outer surface membrane. Glycans bound to the side chain nitrogen atoms of the amino acid aspartic acid residues form N-linked glycoproteins whereas glycans bound to the side chain oxygen atoms of amino acids serine and threonine residues form O-linked glycoproteins.

The finding that almost all protein families expressed on the EV surface membrane, such as integrins, tetraspanins, MMPs and other proteins, are heavily glycosylated [86,87,88], strongly indicates that understanding this glycoprotein pattern is instrumental to decipher the mechanisms by which EVs enter recipient cells [89,90,91]. In other words, surface membrane EV proteomics is essentially glycoproteomics. The main goals of EV glycoproteomics consists of; (a) determining the positions and composition of the entire spectrum of glycans and glycosylated proteins; (b) modifying EV surface membrane glycans by metabolic glycoengineering (MGE) [92,93]. However, although glycoproteomics is likely to shine in the near future as a key tool for unveiling EV-cell interactions, investigating these interactions is not an easy task. There are several hurdles to overcome: first, in some situations, glycans form a wide array of chemical umbrellas covering and obstructing cleavage sites recognized by the proteolytic enzyme used to fragment the backbones of proteins on the EV surface membrane for subsequent MS analysis. Second, glycans include a vast array of isomers that in turn can bind different protein glycosylation sites, thus further complicating the interpretation of MS data output. Third, the complexity of matching MS spectra with database archives requires the development of innovative and dedicated computational applications to run on expensive, high-performance networks or, even better, on cloud systems.

Recent years have witnessed improvements aimed at overcoming these problems: dedicated MS instrumentation together with MS-based approaches, and implementation of new powerful computational solutions for bioinformatics analysis of glycoproteins. The relatively low abundance of certain classes of glycopeptides in EVs compared to whole cells or tissues, requires specific enrichment strategies for the MS-based glycoproteomic assay. Two comprehensive reviews [94,95] describe and comment the most common glycoprotein enrichment techniques, such as lectin arrays and other affinity or interaction methods (HILIC), together with strategies based on recent biological and chemical reagents such as those used in click chemistry.

In the characterization of specific glycosylation sites for glycopeptide analysis, selecting the best beam-type collisional activation in tandem MS is critical for correct interpretation of the results. Another work [96] explored the advantages and disadvantages of several MS-related collision activation protocols for their ability to generate high quality spectra for N- and O-glycopeptides. Although N-glycopeptides are optimally identified by HCD and sceHCD methods, ETD-based methods are more suitable for site-specific analyses of O-glycopeptides; this means that obtaining high quality spectra in N- and O-glycopeptide analysis requires the use of two different dissociation protocols.

A recent work [97] exploits capillary zone electrophoresis coupled with electrospray ionization mass spectrometry (CZE-ESI-MS) to implement a very sensitive platform for the profiling of N-glycans derived from different sources. Notably, this procedure increased detection sensitivity many-fold compared to previous instrument setup, thus allowing the differentiation of several glycan isomers. To improve characterization and functional validation of complex glycoproteins, other authors [98] developed a comparative platform termed SugarQb that allows the identification of the glycosylation pattern and glycan substitutions of proteins from different and complex matrices. SugarQuant [99] is another very innovative tool, specifically aimed at improving the detection of N-glycopeptide fragments. This MS-based platform includes a protein aggregation capture (PAC) system, a dedicated data MS3 acquisition and a processing algorithm (GlycoBinder), thus allowing detailed characterization and precise quantification of a wide array of glycopeptides from complex biological specimens. The characterization of O-glyco-peptides and O-glycans can be achieved by a novel liquid chromatography technique coupled to a tandem mass spectrometry (LC-MS/MS) platform using data-independent acquisition (DIA), termed Glyco-DIA [100]. This system has the great advantage of providing proteome-wide quantification as well as an associated glycosite identification analysis. The huge size and complexity of datasets produced by large-scale glycoprotein and glycopeptide analysis makes the correct interpretations of these data very challenging. In the attempt to speed up analysis and improve glycan-related structure identification, novel search engines have been developed to localize O-glycosites [101] and to identify N- and O-linked glycopeptides. The hope is that their application to the field of EV glycoproteomics could boost research aimed at further proving or disproving the hypothesis of the elusive EV code.

Studies aimed at characterizing the composition of EVs have attempted to pinpoint their primary structures and find a structure-activity correlation. However, the complexity and branching of glycan isoforms, together with their pattern of binding to protein epitopes, makes this a daunting goal. The most practical solution to this problem involves measuring a functional parameter, such EV uptake in recipient cells, after two different treatments that modify EV surface membrane glycan patterns: a subtractive treatment and an additive treatment. The former involves EV treatment with chemical or enzymatic agents that remove N- or O-glycans from surface membrane glycoproteins, to evaluate the ability of these treated EVs to be taken up by recipient cells. Several enzymes with varying specificity are used; PNGase F removes N-linked glycans and cleaves bonds between asparagine and GlcNAc while Endo H and neuraminidase act preferentially on sialic acid-rich glycans. Because no specific enzyme is available to release O-glycans, their removal is often performed by means of chemical agents. However, this latter approach has major pitfalls that could hinder correct interpretation of the results: reactions are usually carried out in very harsh conditions that can damage the integrity of the glycans themselves or other EV surface membrane molecules that could potentially co-modulate the uptake. Despite these drawbacks, several reports have tried to find out a possible correlation between glycan-depleted EVs and their internalization efficiency, tested in different recipient cells. Treatment of mouse liver EVs with neuraminidase, an enzyme that removes the terminal sialic acid residues, increases the uptake in the lung of treated EVs compared to untreated EVs [102]. In addition, treated EVs spread more efficiently in the lymph nodes compared to untreated EVs, thus suggesting that sialic acid glycoproteins play a prominent role in directing their uptake in selected organs. Other authors [103] found that breast cancer cell EXs deprived of surface membrane glycans were uptaken more readily in vivo by human umbilical vein endothelial cells (HUVEC), thus suggesting that glycosylation has a suppressive role in the modulation of EX uptake. The second additive approach is based on protein glycoengineering whose main goal consists of modifying protein to reduce their side-effects and to improve their biophysical and biological properties [79]. This goal can be obtained by two different strategies: (a) biochemistry-glycoengineering based on glycosidase and glycosyltransferase [104,105,106]; and (b) organic chemistry-glycoengineering based on synthetic procedures [107].

Strategy (a) is rather cumbersome and strongly limited by the availability of reliable catalytic enzymes; this pitfall could possibly be circumvented using another class of enzymes, called glycosynthases, that allow a more massive transfer of oligosaccharides to proteins, to give a higher yield [108]. Strategy (b) is more flexible and makes it possible to prepare compounds in which O-linked isoglycoforms are selectively bound to specific amino acids, and thus to study the effect of these modifications on biological parameters [109]. However, the practical downsides of these methods should be taken into careful consideration; the protocols require optimization and standardization, making them suitable only for very experienced organic chemists with well-organized research teams.

These considerations should not be taken as disincentive; although the procedures have mainly been applied to the modification of therapeutic biological molecules such as antibodies or of other recombinant proteins, future developments and refinements are expected to greatly impact on the field of EV glycoproteomics. A slight modification of the additive approach removes and then inserts specific high-affinity glycan-like ligands into EV outer surface membrane molecules to assess changes in their uptake. Indeed, an elegant study [110] has shown that the insertion of a glycoprotein ligand for antigen-presenting cells (APC) into glioblastoma-derived EXs leads to a decrease in immunosuppressive ability accompanied by a concomitant activation of their antitumor immunity. In partial agreement with this result, another report [111] demonstrates that the surface membrane modification of EXs with mannose and PEG could enhance their uptake into dendritic cells and lymph nodes. In another very intriguing report, the authors found that breast cancer cells release parental EVs with reduced bisecting GlcNAc [112], which could enhance the metastatic potential of recipient cells. Quite interestingly, metastatic potential was diminished in EVs bearing a high bisecting GlcNAc modification in the vesicular integrin β1, thus demonstrating that specific surface membrane glycoprotein, such as integrins, play a key role in EV uptake in target cells. 

### Metabolic Glycoengineering of Extracellular Vesicles

Metabolic glycoengineering (MGE) is based on the incorporation into the EV structure of a synthetic modified monosaccharide, structurally similar to a natural precursor but bearing an unnatural chemical group. The necessary requirement is that the modified monosaccharide be processed by a biosynthetic pathway involved in the formation of a membrane glycan bearing the unnatural functional group [113]. The application of MGE to EVs is a giant leap forward in the development of methods aimed at separating and then analyzing the expression pattern of EVs and cell surface membrane glycoproteins [114,115]. The basic protocol includes the preliminary separation and enrichment of surface membrane labeled glycoproteins via click chemistry and affinity chromatography methods, followed by site-specific peptide characterization by HPLC/MS-MS. Recent studies have greatly emphasized the key role of surface membrane glycans in the regulation of cell-cell communications in oncology; cancer cells with high invasiveness and metastatic properties display an overexpression of N-sialo-glycoproteins belonging to an epitope localized on cell-adhesion domains. The combination of MGE with bio-orthogonal click chemistry bears extraordinary advantages over other reactions; indeed, click chemistry is very quick, highly specific, proceeds under physiological conditions and, notably, several recent improvements [116,117] could bypass the toxicity related to copper catalysis that can damage live cells or interfere with EV functionality. Although in theory, MGE is a relatively easy and straightforward technique, some conditions and parameters of modified precursor incubation need careful consideration and optimization. These variable factors include the concentration and incubation time of precursors necessary to ensure a detectable expression on the EV surface; the tendency of cells to uptake the precursors, and the turnover rate and metabolism of modified glycans. Another problem could arise from the heterogeneity of glycan structures and the associated complexity and redundancy of the glycan metabolic pathway that can lead to the formation of glycan isomers. 

Another approach, based on the addition of click chemistry groups, consists of a two-step protocol [118]. Purified EXs were first cross-linked with alkyne groups to primary amines of surface membrane proteins and then EXs were conjugated with azide-fluor545 via copper-catalyzed azide–alkyne cyclo-addition. This method does not modify either the size of EXs or their extent of docking or internalization into recipient cells, and may therefore be a suitable and reproducible tool to add alkyne residues to EV surfaces for further EV structural and localization experiments. One limitation of this methodology is the presence of copper-like catalysts, which may damage EV viability; however, this drawback could be elegantly bypassed by procedures based on copper-free click chemistry. In another study, ref [119] EXs isolated from different cell lines were treated with the DBCO-NHS (dibenzo-cyclooctyne-N-hydroxy-succinimidyl ester) reagent; the NHS group binds the primary amines of proteins while the exposed DBCO group allows cross-linking to azides via copper-free click chemistry. The incubation of modified EXs with azide-alexafluor488 led to the formation of dye-labeled EXs that can be used to monitor EX docking, intracellular tracking, and extent of uptake in target cells.

## 5. Analysis of Single Extracellular Vesicles

Although the intercellular heterogeneity of EVs has been clearly documented, their intracellular heterogeneity is still open to speculation due to the paucity of experimental data. Unfortunately, almost all studies so far published have carried out bulk analyses of EV proteins, masking individual EV expression profiles and leading to erroneous conclusions. Indeed, at present, the influence on cell culture models of parameters such as external stimuli, proliferation, motility, and cell density on the heterogeneity of released EVs is not known. 

Efforts to overcome this issue have stimulated attempts to set up platforms devoted to analyzing surface membrane patterns at the single EV level [120]. Recently, an innovative and extremely sensitive proximity-dependent barcoding assay, using antibody-DNA conjugates coupled with next-generation sequencing, was implemented to profile the surface membrane protein patterns of single EVs [121]. The authors reported that this assay was able to measure the expression of 38 different proteins in human body fluids or cell culture-derived EXs, thus strengthening the notion that the identification of EV surface membrane protein patterns is a reliable tool to distinguish EVs derived from different sources. Multiplexed single EV protein profiling has been implemented in another similar procedure that combines sequentially EV labeling with barcoded antibody-DNA in a microfluidic device, followed by immuno-droplet digital polymerase chain reaction (iddPCR) [122]. When glioma cell-derived EVs were tested, the authors found that a considerable fraction of droplets were positive for only one marker (i.e., EGFR or EpCAM) but a discrete percentage (39%) of these EVs displayed positivity for both markers. Although in comparison with traditional massive MS analysis, these two approaches detect a smaller number of proteins and require DNA-barcoded antibodies coupled to microfluidic systems, their excellent sensitivity paves the way for the development of future platforms in single EV analysis.

A tool combining separation of EVs based on their size with individual surface membrane protein analysis by in situ fluorescence labeling, termed EV-Ident [123], found a difference in expression of certain markers between breast cancer and prostate cancer cell-derived EVs. Notably, this study allows the determination of EV markers in EVs from separate sources based on their size, thus expanding our knowledge of medium and large EVs, which are usually poorly investigated in other reports. Another approach based on localized fluorescent imaging, termed digital profiling of proteins on individual EV (DPPIE), uses an anti-CD9 antibody engineered biochip to capture EVs from clinical plasma samples and then a multiple DNA aptamer coupled to rolling circle amplification (RCA) to generate localized fluorescent signals [124]. This method, beyond providing several improvements over existing bulk measurements such as ELISA, found that CD63/EpCAM/MUC1-triple-positive EVs are significantly more numerous in breast cancer patients than in healthy donors. Notably, this method was able to discriminate between EVs derived from lung adenocarcinoma and lung squamous carcinoma patients.

The strong limitation of having to detecting tiny amounts of proteins localized on single EVs has been partially overcome by the combination of FACS analysis with signal amplification methods. Indeed, the implementation of FACS-assisted multiplex and multicolor in situ proximity ligation assay (in situ PLA) together with multiplex proximity extension assays (PEA), was instrumental in the precise assessment of EV surface membrane protein patterns and in the classification of EVs into subpopulations [125,126]. In another almost identical approach [127], EVs were immobilized using a biotin-avidin capture system and labeled with probes. Positive EVs were detected by fluorescence imaging. This assay reveals not only the diversity of EVs but also distinct tetraspanine expression patterns. Finally, to better stratify GBM-derived EVs, another team [128] implemented a very sensitive, multiplexable method termed SEA (single EV analysis). In this platform EVs are first immobilized using a biotin-streptavidin system and then further characterized by immuno-fluorescence with antibodies directed against a panel of 11 different specific markers including EpCam and EGFR-variant III. This study revealed some interesting properties of examined EVs: (a) about half of EVs only expressed one or two of the 11 markers evaluated; (b) only a small percentage of EVs exhibited five or more markers; (c) four EV marker subtypes accounted for most of the total population and (d) cluster analysis identified 14 main populations based on marker expression. SEA analysis found that 366 proteins out of a total 2178 were consistently present at higher levels in EVs than in cells, and that EXs mostly express CD9, whereas 50% of large EVs are either CD9 or CD81 positive [129]. In addition, an analysis of single EVs derived from GBM cell lines or plasma of GBM patients [130] disclosed several interesting features of MVs including the expression of tetraspanins together with the unexpected observation that tumoral MVs represent about 10% of total plasma MVs in GBM patients. One major limitation of the SEA approach resides in the lack of a standardized method to establish a valid cutoff for a particular marker to differentiate between true negative EVs and false negative EVs expressing the same marker but below the assay threshold. Once the improvements in new technologies overcome this pitfall, we foresee that SEA technology will become a useful tool in basic EV biology and in clinical settings.

## 6. EVs and Vaccines

The SARS-CoV-2 virus uses the spike proteins sticking out of its surface to attach to and enter cells in the human body. These proteins are coated with glycans, which disguise parts of the viral proteins to the human immune system. Chemical analysis of the glycans coating the spike protein [131] will be critical in the development of new COVID-19 vaccines that rely on recombinant spike protein [132] triggering the immune response.

The similarities between viruses and EVs have prompted efforts by a drug company to use EXs in vaccine development [133]. Indeed, this company is exploiting the intriguing hypothesis that EXs could be a more efficient and safer delivery vehicle for mRNA than the currently used lipid nanoparticles (LNPs). This fast-growing field, still in its early infancy, has generated some interesting questions: (a) are EVs a better shuttle for mRNA delivery than LNP, modified or attenuated viruses?; (b) do cells infected with conventional vaccines release EVs bearing on their surface the exogenous protein codified by mRNA?; (c) is it possible to modify, chemically or by metabolic engineering, the composition of surface membrane EV glycoproteins to enhance their uptake in recipient cells?; and last but not least (d) could modified EVs bearing on their surface the protein(s) used by the virus to gain cell access, be used to trigger an immune response or compete with viruses to bind to cell entry receptors?

## 7. Future Trends

**Figure 1:**
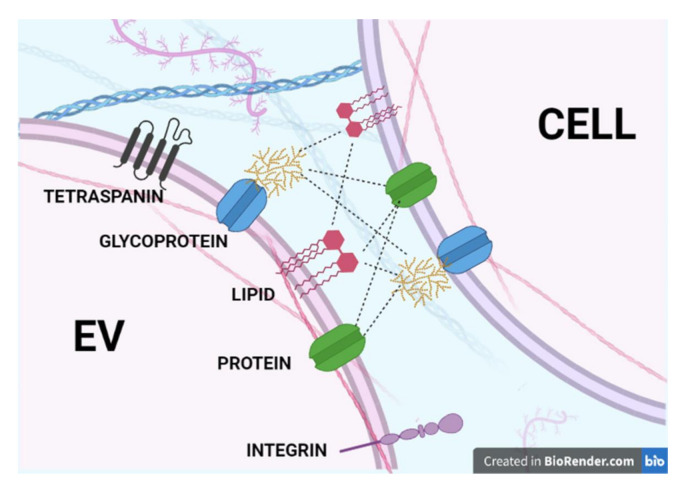
Possible interactions between EV membrane surface molecules and their counterparts in recipient cells. Dashed lines indicate all possible combinations of putative interactions. Protein include any PTM modifications.

Recent advances in the identification of EVs and cell glycoproteins, together with methods for screening their reciprocal interactions, have greatly expanded our knowledge of EV-cell interaction [134]. Unfortunately, none of the approaches applied so far has been able to conclusively pinpoint the relationships occurring at molecular level during EV docking with recipient cells. The problem is that the assays usually detect in vitro affinity between isolated or purified molecules and immobilized probes in experimental conditions that are very far from the membrane physiological context [135]. Indeed, the pattern of possible reciprocal interactions between EV outer surface membrane components and their cellular counterparts is quite complex and so far insufficiently investigated (Figure 1). Therefore, in our modest opinion, defining EV-cell interactions at the molecular level requires the further development of innovative technologies based on the combination of three different already-existing chemical methodologies: cross-linking, ligand-receptor capture assay (LRC) and click chemistry. Problems associated with the molecular-level study of interactions at the cell surface have recently been exploited by means of an elegant combined chemo-proteomic technique [136]. HATRIC-based ligand-receptor capture (HATRIC-LRC) is a powerful, innovative technology developed to characterize living cell target receptors for a wide variety of ligands, including endogenous/exogenous peptides acting as agonists/antagonists, small molecules, and viruses. HATRIC-LRC technology exploits the chemical reactivity of the trifunctional cross-linker HATRIC, which bears three different arms: (1) a NHS-ester to attach the ligand to be tested via reaction with primary amines; (2) a 5-MA (5-methoxyanthranilic acid) hydrazone to covalently bind previously oxidized glycans of the unknown target proteins at the cell membrane; and (3) an azide group to pull down the formed tripartite complex by a classical azide–alkyne click chemistry reaction. Briefly, the procedure starts with mild oxidation of the glycans present on the surface membrane of cells expressing the unknown target, followed by addition of the HATRIC-ligand. The first NHS-ester arm targets proteins on the cell membrane; the second arm covalently binds to the oxidized glycans; and then, after cell lysis, the third arm of HATRIC pulls down the tripartite complex. The complex is treated with trypsin or other proteases and proteins are identified by bottom-up MS-proteomics. The HATRIC-LRC technology platform requires a relatively small number of cells (about 1 million), compared to the previously implemented LRC-TriCEPS method [137,138] and, in addition, it allows the identification of links between HATRIC-bound ligands with surface N- and O-glycosylated proteins under physiological conditions that do not alter cell viability.

At this point an intriguing question arises: could an analogous platform be devised and chemically engineered to pinpoint interactions between EVs and target cells at molecular level? A working hypothesis may consist of synthesizing a trifunctional ligand (TC) with an arm bearing a residue for affinity purification (such as biotin) and the other two arms bearing similar or different click chemistry reagents. This TC could be incubated with previously modified EVs or cells, metabolically or chemically labeled with complementary, matching click chemistry reagents or other compatible residues, thus allowing the TC to form a bridge between interacting molecules. The TC-ligand complex will then be purified by the separating arms, treated with proteases and/or glycosidase and finally analyzed by HPLC-MS. This latter technique should theoretically allow, following spectral interpretation, correct assignment of EVs or target cells glycoproteins.

Interestingly, similar platforms have already been used to characterize the docking of soluble protein ligands [139]; to target small-molecule drugs to receptors using tetrafunctional probes [140] and finally in protein labeling and capture using cleavable trifunctional reagents [141]. Should such an approach be implemented, two critical issues will need to be addressed (a) the trifunctional ligand reacts with aldehydes derived from oxidized outer surface membrane glycans, and this oxidation step may reduce assay specificity thus masking “true” physiological interactions; (b) kinetic studies will have to be carefully planned because the trifunctional reagent complex could be rapidly internalized and cleaved by endogenous proteases thus leading to misinterpretation of glycoprotein structures.

Finally, a recent study [142] has shown that some N-glycosylated members of the small non-coding RNA family are displayed on the outer surface of several cell types. These glycoRNAs appear to play an important functional role in cell-adhesion mechanisms because they can interact with multiple members of the Siglec receptor family. Quite notably, these small non-coding RNAs, such as the family of Y RNAs, are enriched in the extracellular RNAs supernatant fraction and in EX RNAs derived from cultured human glioma stem cells (GSCs) [143]. Future studies are therefore warranted to ascertain whether these newly identified glycoRNAs, which would constitute a new piece in the EV delivery code puzzle, are expressed on the outer membrane surface of EVs.

## 8. Conclusions

In conclusion, do EVs possess a delivery code? The verdict is not out yet: there are some tantalizing findings, but they are still scarce and sometimes results have not been properly replicated or confirmed in other studies. Many more experiments, using the most recent and sophisticated analytical and structural techniques, are required. We foresee that in the near future, the main progress in cracking the putative EV delivery code will emerge from two different but complementary approaches.

The first is the synthesis of chemically modified metabolic precursors [144] to widen the click chemistry armamentarium thus making available highly reactive probes and reporters. These new reagents will have to be compatible with in vivo and in vitro platforms and their incorporation should bring reactive residues on EV and the outer surface of cell membranes.

The second, theoretical and bioinformatic, will depend strictly on the implementation of more and more sophisticated and powerful software, such as the recent ground-breaking algorithm Alpha Fold, which can infer the folding of PTM proteins and other surface membrane molecules.

This open verdict translates into a compelling call to the vast community of chemists, cell biologist and glycol-proteomics scientists to combine their valuable contributions and efforts to participate in a multidisciplinary project to work on unveiling EV-cell and cell-cell interaction codes. The challenge is very daunting, but the potential outcomes will be of seminal importance for the future of pharmacology and medicine.

## Figures and Tables

**Table 1:** Programs for the analysis of membrane topology domains.

PROGRAM.	FUNCTION	URL	Ref
EXPASY	over 160 databases and software tools for life science and clinical research	expasy.org, accessed on 3 June 2021	[35]
MOLBIOTOOLS	Collection of free online apps for molecular biology	molbiotools.com, accessed on 8 July 2021	
MEMBRANOME 2.0	provides structural and functional data about single-spanning (bitopic) transmembrane proteins	membranome.org, accessed on 8 July 2021	[36]
PREDICT PROTEIN 2013	predictions of regular and non-regular secondary structure and intrinsically disordered regions	predictprotein.org, accessed on 8 July 2021	[37]
PROTTER	interactive integration and visualization of annotated and predicted protein sequence	wlab.ethz.ch/protter/start, accessed on 8 July 2021	[38]
SCRATCH PROTEIN PREDICTOR	secondary structure prediction based on protein evolutionary information	scratch.proteomics.ics.uci.edu, accessed on 8 July 2021	[39]
SURFY	in silico human surfaceome	wlab.ethz.ch/surfaceome, accessed on 8 July 2021	[40]
TMHMM	prediction of transmembrane helices in proteins	cbs.dtu.dk/services/TMHMM, accessed on 8 July 2021	[41]
TOPCONS	Consensus prediction of membrane protein topology and signal peptides	topcons.cbr.su.se, accessed on 8 July 2021	[42]
YASPIN	HMM-based neural network secondary structure prediction using PSI-BLAST PSSM matrices	ibi.vu.nl/programs, accessed on 8 July 2021	[43]

## Abbreviations

ACE: angiotensin converting enzyme; APC: antigen-presenting cells; BBB: blood brain barrier; CuAAC: copper-catalyzed azide–alkyne cycloaddition; CZE-ESI-MS: capillary zone electrophoresis coupled to electrospray ionization mass spectrometry; DBCO-NHS: dibenzo cyclooctyne-N-hydroxysuccinimidyl ester; DIA: data-independent acquisition; DPPIE: digital profiling of proteins on individual EV; EGFR: epidermal growth factor receptor; ELISA: enzyme linked immunosorbent assay; EpCAM: epithelial cell-adhesion molecule; ETD: electron transfer dissociation; EV: extracellular vesicle; EX: exosome; FACS: fluorescence-activated cell sorting FSO_2_N_3_: fluor sulfuryl azide; GBM: glioblastoma; GlcNAc: β-N-acetylglucosamine; HATRIC-LRC: HATRIC-based ligand-receptor capture; HCD: higher-energy collisional dissociation; HILIC: hydrophilic interaction liquid chromatography; HPLC-MS: high pressure liquid chromatography-mass spectrometry; HSPGs: heparan sulfate proteoglycans; HUVEC: human umbilical vein endothelial cells; IC: immunoaffinity capture; iddPCR: immuno-droplet digital polymerase chain reaction; LC-MS/MS: liquid chromatography coupled to tandem mass spectrometry; LNP: lipid nanoparticle; LRC: ligand-receptor capture assay; 5-MA: 5-methoxyanthranilic acid; MGE: metabolic glycoengineering; MPP: matrix metalloprotease; MV: micro vesicle; NHS: N-Hydroxysuccinimidyl esters; NTA: nanoparticle tracking analysis; PAC: protein aggregation capture; PDAC: pancreatic ductal adenocarcinoma; PEA: proximity extension assay; PEG: polyethylenglycol; PLA: proximity ligation assay; PNGase F: N-glicosidasi F; PTM: post-translation modification; RCA: rolling circle amplification; sceHCD: stepped collision energy HCD; higher-energy collisional dissociation; SEA: single EV analysis; SEC: size-exclusion chromatography; SWATH-MS: sequential window acquisition of all theoretical mass spectra; TC: trifunctional compound; UC: ultracentrifugation.

## References

[1] Abels E.R., Brakefield X.O. (2016). Introduction to Extracellular Vesicles: Biogenesis, RNA Cargo Selection, Content, Release, and Uptake. Cell Mol. Neurobiol..

[2] van Niel G., D’Angelo G., Raposo G. (2018). Shedding light on the cell biology of extracellular vesicles. Nat. Rev. Mol. Cell Biol..

[3] Margolis L., Sadovsky Y. (2019). The biology of extracellular vesicles: The known unknowns. PLoS Biol..

[4] Negahdaripour M., Owji H., Eskandari S., Zamani M., Vakili B., Nezafat N. (2021). Small extracellular vesicles (sEVs): Discovery, functions, applications, detection methods and various engineered forms. Expert Opin. Biol. Ther..

[5] Tkach M., Thery C. (2016). Communication by Extracellular Vesicles: Where We Are and Where We Need to Go. Cell.

[6] Wu P., Zhang B., Ocansey D.K.W., Xu W., Qian H. (2021). Extracellular vesicles: A bright star of nanomedicine. Biomaterials.

[7] Jadli A.S., Ballasy N., Edalat P., Patel V.B. (2020). Inside(sight) of tiny communicator: Exosome biogenesis, secretion, and uptake. Mol. Cell Biochem..

[8] Greening D.W., Simpson R.J. (2018). Understanding extracellular vesicle diversity—Current status. Expert Rev. Proteom..

[9] Ailuno G., Baldassari S., Lai F., Florio T., Caviglioli G. (2020). Exosomes and Extracellular Vesicles as Emerging Theranostic Platforms in Cancer Research. Cells.

[10] Panfoli I., Bruschi M. (2020). The good and bad sides of exosomes: Pre-metastatic niche formation, cancer biomarker and therapy carriers. J. Cancer Metastasis Treat..

[11] Maas S.L.N., Breakefield X.O., Weaver A.M. (2017). Extracellular vesicles: Unique intercellular delivery vehicles. Trends Cell Biol..

[12] Zappulli V., Friis K.P., Fitzpatrick Z., Maguire C.A., Breakefield X.O. (2016). Extracellular vesicles and intercellular communication within the nervous system. J. Clin. Investig..

[13] Mulcahy L.A., Pink R.C., Carter D.R. (2014). Routes and mechanisms of extracellular vesicle uptake. J. Extracell. Vesicles.

[14] French K.C., Antonyak M.A., Cerione R.A. (2017). Extracellular vesicle docking at the cellular port: Extracellular vesicle binding and uptake. Semin. Cell Dev. Biol..

[15] Martinelli C., Gabriele F., Dini E., Carriero F., Bresciani G., Slivinschi B., Giudici M.D., Zanoletti L., Manai F., Paolillo M. (2020). Development of Artificial Plasma Membranes Derived Nanovesicles Suitable for Drugs Encapsulation. Cells.

[16] Wang J., Li W., Lu Z., Zhang L., Hu Y., Li Q., Du W., Feng X., Jia H., Liu B.-F. (2017). The use of RGD-engineered exosomes for enhanced targeting ability and synergistic therapy toward angiogenesis. Nanoscale.

[17] Zheng M., Huang M., Ma X., Chen H., Gao X. (2019). Harnessing Exosomes for the Development of Brain Drug Delivery Systems. Bioconjug. Chem..

[18] Hoen E.N., Cremer T., Gallo R.C., Margolis L.B. (2016). Extracellular vesicles and viruses: Are they close relatives?. Proc. Natl. Acad. Sci. USA.

[19] van Dongen H.M., Masoumi N., Witwer K.W., Pegtel D.M. (2016). Extracellular Vesicles Exploit Viral Entry Routes for Cargo Delivery. Microbiol. Mol. Biol. Rev..

[20] Mori T., Jung J., Kobayashi C., Dokainish H.M., Re S., Sugita Y. (2021). Elucidation of interactions regulating conformational stability and dynamics of SARS-CoV-2 S-protein. Biophys. J..

[21] Hou R., Li Y., Sui Z., Yuan H., Yang K., Liang Z., Zhang L., Zhang Y. (2019). Advances in exosome isolation methods and their applications in proteomic analysis of biological samples. Anal. Bioanal. Chem..

[22] Doyle L.M., Wang M.Z. (2019). Overview of Extracellular Vesicles, Their Origin, Composition, Purpose, and Methods for Exosome Isolation and Analysis. Cells.

[23] Rai A., Fang H., Fatmous M., Claridg B., Poh Q.H., Simpson R.J., Greening D.W. (2021). A Protocol for Isolation, Purification, Characterization, and Functional Dissection of Exosomes. Methods Mol. Biol..

[24] Gandham S., Su X., Wood J., Nocera A.L., Alli S.C., Milane L., Zimmerman A., Amiji M., Ivanov A.R. (2020). Technologies and Standardization in Research on Extracellular Vesicles. Trends Biotechnol..

[25] Zhang Y., Bi J., Huang J., Tang Y., Du S., Li P. (2020). Exosome: A Review of Its Classification, Isolation Techniques, Storage, Diagnostic and Targeted Therapy Applications. Int. J. Nanomed..

[26] Chen Y., Zhu Q., Cheng L., Wang Y., Li M., Yang Q., Hu L., Lou D., Li J., Dong X. (2021). Exosome detection via the ultrafast-isolation system: EXODUS. Nat. Methods.

[27] Kim D., Kang B., Kim O.Y., Choi D., Lee J. (2013). EVpedia: An integrated database of high-throughput data for systemic analyses of extracellular vesicles. J. Extracell. Vesicles.

[28] Pathan M., Fonseka P., Chitti S.V., Kang T., Sanwlani R., Van Deun J., Hendrix A., Mathivanan S. (2019). Vesiclepedia 2019: A compendium of RNA, proteins, lipids and metabolites in extracellular vesicles. Nucleic Acids Res..

[29] Keerthikumar S., Chisanga D., Ariyaratne D., Al Saffar H., Anand S., Zhao K., Samuel M., Pathan M., Jois M., Chilamkurti N. (2016). ExoCarta: A web-based compendium of exosomal cargo. J. Mol. Biol..

[30] Van Deun J., Mestdagh P., Agostinis P., Akay Ö., Anand S., Anckaert J., Martinez Z.A., Baetens T., Beghein E., Bertier L. (2017). EV-TRACK: Transparent reporting and centralizing knowledge in extracellular vesicle research. Nat. Methods.

[31] http://www.microvesicles.org/.

[32] http://www.exocarta.org/.

[33] van Geest M., Lolkema J.S. (2000). Membrane Topology and Insertion of Membrane Proteins: Search for Topogenic Signals. Microbiol. Mol. Biol. Rev..

[34] Lee H., Kim H. (2014). Membrane topology of transmembrane proteins: Determinants and experimental tools. Biochem. Biophys. Res. Commun..

[35] Gasteiger E., Gattiker A., Hoogland C., Ivanyi I., Appel R.D., Bairoch A. (2003). ExPASy: The proteomics server for in-depth protein knowledge and analysis. Nucleic Acids Res..

[36] Lomize A.L., Hage J.M., Pogozheva I.D. (2018). Membranome 2.0: Database for proteome-wide profiling of bitopic proteins and their dimers. Bioinformatics.

[37] Radivojac P., Clark W.T., Oron T.R., Schnoes A.M., Wittkop T. (2013). A large-scale evaluation of computational protein function prediction. Nat. Methods.

[38] Omasits U., Ahrens C.H., Müller S., Wollscheid B. (2014). Protter: Interactive protein feature visualization and integration with experimental proteomic data. Bioinformatic.

[39] Cheng J., Randall A.Z., Sweredoski M.J., Baldi P. (2005). SCRATCH: A protein structure and structural feature prediction server. Nucleic Acids Res..

[40] Bausch-Fluck D., Goldmann U., Müller S., van Oostrum M., Müller M., Schubert O.T., Wollscheid B. (2018). The in silico human surfaceome. Proc. Natl. Acad. Sci. USA.

[41] Krogh A., Larsson B., von Heijne G., Sonnhammer E.L. (2001). Predicting transmembrane protein topology with a hidden Markov model: Application to complete genomes. J. Mol. Biol..

[42] Tsirigos K.D., Peters C., Shu N., Käll L., Elofsson A. (2015). The TOPCONS web server for consensus prediction of membrane protein topology and signal peptides. Nucleic Acids Res..

[43] Lin K., Simossis V.A., Taylor W.R., Heringa J. (2005). A simple and fast secondary structure prediction method using hidden neural networks. Bioinformatics.

[44] Besingi R.N., Clark P.L. (2015). Extracellular protease digestion to evaluate membrane protein cell surface localization. Nat. Protoc..

[45] Olaya-Abril A., Jiemez-Munguìa I., Gomez-Gascoòn L., Rodriguez-Ortega M.J. (2014). Surfomics: Shaving live organisms for a fast proteomic identification of surface proteins. J. Proteom..

[46] Siciliano R.A., Lippolis R., Mazzeo M.F. (2019). Proteomics for the Investigation of Surface-Exposed Proteins in Probiotics. Front. Nutr..

[47] Skliar M., Chernyshev V., Belnap D.M., Sergey G.V., Al-Hakami S.M., Bernard P.S., Stijleman I.J., Rachamadugu R. (2018). Membrane proteins significantly restrict exosome mobility. Biochem. Biophys. Res. Commun..

[48] Cvjetkovic A., Jang S.C., Konečná B., Höög J.L., Sihlbom C., Lässer C., Lötvall J. (2016). Detailed Analysis of Protein Topology of Extracellular Vesicles–Evidence of Unconventional Membrane Protein Orientation. Sci. Rep..

[49] Meng G., Guo T., Ma T., Zhang J., Shen Y., Sharpless K.B., Dong J. (2019). Modular click chemistry libraries for functional screens using a diazotizing reagent. Nature.

[50] Gonda A., Kabagwira J., Senthil G.N., Wall N.R. (2019). Internalization of Exosomes through Receptor-Mediated Endocytosis. Mol. Cancer Res..

[51] Buzás E.I., Tóth E.Á., Sódar B.W., Szabó-Taylor K.E. (2018). Molecular interactions at the surface of extracellular vesicles. Semin. Immunopathol..

[52] Mathieu M., Martin-Jaular L., Lavieu G., Théry C. (2019). Specificities of secretion and uptake of exosomes and other extracellular vesicles for cell-to-cell communication. Nat. Cell Biol..

[53] Horibe S., Tanahashi T., Kawauchi S., Murakami Y., Rikitake Y. (2018). Mechanism of recipient cell-dependent differences in exosome uptake. BMC Cancer.

[54] Lee T.H., Chennakrishnaiah S., Meehan B., Montermini L., Garnier D., D’Asti E., Hou W., Magnus N., Gayden T., Jabado N. (2016). Barriers to horizontal cell transformation by extracellular vesicles containing oncogenic H-ras. Oncotarget.

[55] Fitzner D., Schnaars M., van Rossum D., Krishnamoorthy G., Dibaj P., Bakhti M., Regen T., Hanisch U.-K., Simons M. (2011). Selective transfer of exosomes from oligodendrocytes to microglia by micropinocytosis. J. Cell Sci..

[56] Montecalvo A., Larregina A.T., Shufesky W.J., Stolz D.B., Sullivan M.L.G., Karlsson J.M., Baty C.J., Gibsom G.A., Erdos G., Wang Z. (2012). Mechanism of transfer of functional microRNAs between mouse dendritic cells via exosomes. Blood.

[57] Prada I., Amin L., Furlan R., Legname G., Verderio C., Cojoc D. (2016). A new approach to follow a single extracellular vesicle-cell interaction using optical tweezers. Biotechniques.

[58] Feng D., Zhao W.-L., Ye Y.-Y., Bai X.-C., Liu R.-Q., Chang L.-F., Zhou Q., Sui S.-F. (2010). Cellular internalization of exosomes occurs through phagocytosis. Traffic.

[59] Lai C.P., Mardini O., Ericsson M., Prabhakar S., Maguire C.A., Chen J.W., Tannous B.A., Breakefield X.O. (2014). Dynamic biodistribution of extracellular vesicles in vivo using a multimodal imaging reporter. ACS Nano.

[60] Wiklander O.P.B., Nordin J., O’Loughlin A., Gustafsson Y., Corso G., Mäger I., Vader P., Lee Y., Sork H., Seow Y. (2015). Extracellular vesicle in vivo biodistribution is determined by cell source, route of administration and targeting. J. Extracell. Vesicles.

[61] Peinado H., Aleckovic M., Lavotshkin S., Matei I., Costa-Silva B., Moreno-Bueno G., Hergueta-Redondo M., Williams C., Garcia-Santos G., Ghajar C.M. (2012). Melanoma exosomes educate bone marrow progenitor cells toward a pro-metastatic phenotype through MET. Nat. Med..

[62] Nieland L., Morsett L.M., Broekman M.L.D., Breakefield X.O., Abels E.R. (2021). Extracellular Vesicle-Mediated Bilateral Communication between Glioblastoma and Astrocytes. Trends Neurosci..

[63] You Y., Borgmann K., Edara V.V., Stacy S., Ghorpade A., Ikezu T. (2020). Activated human astrocyte-derived extracellular vesicles modulate neuronal uptake, differentiation and firing. J. Extracell. Vesicles.

[64] Chivet M., Javalet C., Laulagnier K., Blot B., Hemming F.J., Sadoul R. (2014). Exosomes secreted by cortical neurons upon glutamatergic synapse activation specifically interact with neurons. J. Extracell. Vesicles.

[65] Hoshino A., Costa-Silva B., Shen T.-L., Rodrigues G., Hashimoto A., Mark M.T., Molina H., Kohsaka S., Di Giannatale A., Ceder S. (2015). Tumour exosome integrins determine organotropic metastasis. Nature.

[66] Paolillo M., Galiazzo M.C., Daga A., Ciusani E., Serra M., Colombo L., Schimelli S. (2018). An RGD small-molecule integrin antagonist induces detachment-mediated anoikis in glioma cancer stem cells. Int. J. Oncol..

[67] Paolillo M., Colombo R., Serra M., Belvisi L., Papetti A., Ciusani E., Comincini S., Schinelli S. (2019). Stem-Like Cancer Cells in a Dynamic 3D Culture System: A Model to Study Metastatic Cell Adhesion and Anti-Cancer Drugs. Cells.

[68] Paolillo M., Schinelli S. (2017). Integrins and Exosomes, a Dangerous Liaison in Cancer Progression. Cancers.

[69] Grigoryeva E.S., Savelieva O.E., Popova N.O., Cherdyntseva N.V., Perelmuter V.M. (2020). Do tumor exosome integrins alone determine organotropic metastasis?. Mol. Biol. Rep..

[70] DeRita R.M., Sayeed A., Garcia V., Krishn S.R., Shields C.D., Sarker S., Friedman A., McCue P., Molugu S.K., Rodeck U. (2019). Tumor-Derived Extracellular Vesicles Require β1 Integrins to Promote Anchorage-Independent Growth. iScience.

[71] Christianson H.C., Svensson K.J., van Kuppevelt T.H., Li J., Belting M. (2013). Cancer cell exosomes depend on cell-surface heparan sulfate proteoglycans for their internalization and functional activity. Proc. Natl. Acad. Sci. USA.

[72] Wu A.Y., Ueda K., Lai C.P. (2019). Proteomic Analysis of Extracellular Vesicles for Cancer Diagnostics. Proteomics.

[73] Castillo J., Bernard V., Lucas F.A.S., Allenson K., Capello M., Kim D.U., Gascoyne P., Mulu F.C., Stephens B.M., Huang J. (2018). Surfaceome profiling enables isolation of cancer-specific exosomal cargo in liquid biopsies from pancreatic cancer patients. Ann. Oncol..

[74] Mathivanan S., Lim J.W.E., Tauro B.J., Ji H., Moritz R.L., Simpson R.J. (2010). Proteomics Analysis of A33 Immunoaffinity- purified Exosomes Released from the Human Colon Tumor Cell Line LIM1215 Reveals a Tissue-specific Protein Signature. Mol. Cell. Proteom..

[75] Kowal J., Arras G., Colombo M., Jouve M., Morath J.P., Primdal-Bengtson B., Dingli F., Loew D., Tkach M., Théry C. (2016). Proteomic comparison defines novel markers to characterize heterogeneous populations of extracellular vesicle subtypes. Proc. Natl. Acad. Sci. USA.

[76] HHoshino A., Kim H.S., Bojmar L., Gyan K.E., Cioffi M., Hernandez J., Zambirinis C.P., Rodrigues G., Molina H., Heissel S. (2020). Extracellular Vesicle and Particle Biomarkers Define Multiple Human Cancers. Cell.

[77] Chutipongtanate S., Greis K.D. (2018). Multiplex Biomarker Screening Assay for Urinary Extracellular Vesicles Study: A Targeted Label-Free Proteomic Approach. Sci. Rep..

[78] Novikova S., Shushkova N., Farafonova T., Tikhonova O., Kamyshinsky R., Zgoda V. (2020). Proteomic Approach for Searching for Universal, Tissue-Specific, and Line-Specific Markers of Extracellular Vesicles in Lung and Colorectal Adenocarcinoma Cell Lines. Int. J. Mol. Sci..

[79] Lara P., Chan A.B., Cruz L.J., Quest A.F.G., Kogan M.J. (2020). Exploiting the Natural Properties of Extracellular Vesicles in Targeted Delivery towards Specific Cells and Tissues. Pharmaceutics.

[80] Ma B., Guan X., Li Y., Shang S., Li J., Tan Z. (2020). Protein glycoengineering: An approach for improving protein properties. Front. Chem..

[81] Zhu Q., Heon M., Zhao Z., He M. (2018). Microfluidic engineering of exosomes: Editing cellular messages for precision therapeutics. Lab Chip.

[82] Salunkhe S., Dheeraj, Basak M., Chitkara D., Mittal A. (2020). Surface functionalization of exosomes for target-specific delivery and in vivo imaging & tracking: Strategies and significance. J. Control. Release.

[83] Tian T., Zhang H.-X., He C.-P., Fan S., Zhu Y.-L., Qi C., Huang N.-P., Xiao Z.-D., Lu Z.-H., Tannous B.A. (2018). Surface Functionalized Exosomes as Targeted Drug Delivery Vehicles for Cerebral Ischemia Therapy. Biomaterials.

[84] Smith B.A., Bertozzi C.R. (2021). The clinical impact of glycobiology: Targeting selectins, Siglecs and mammalian glycans. Nat. Rev. Drug Discov..

[85] Gabius H.J. (2018). The sugar code: Why glycans are so important. BioSystems.

[86] Martins Á.M., Ramos C.C., Freitas D., Reis C.A. (2021). Glycosylation of Cancer Extracellular Vesicles: Capture Strategies, Functional Roles and Potential Clinical Applications. Cells.

[87] Gerlach J.Q., Griffin M.D. (2016). Getting to know the extracellular vesicle glycome. Mol. Biosyst..

[88] Williams C., Royo F., Aizpurua-Olaizola O., Pazos R., Boons G.-J., Reichardt N.-C., Falcon-Perez J.M. (2018). Glycosylation of extracellular vesicles: Current knowledge, tools and clinical perspectives. J. Extracell. Vesicles..

[89] Williams C., Pazos R., Royo F., González E., Roura-Ferrer M., Martinez A., Gamiz J., Reichardt N.-C., Falcón-Pérez J.M. (2019). Assessing the role of surface glycans of extracellular vesicles on cellular uptake. Sci. Rep..

[90] Liang Y., Eng W.S., Colquhoun D.R., Dinglasan R., Graham D.R., Mahal L.K. (2014). Complex N-Linked Glycans Serve as a Determinant for Exosome/Microvesicle Cargo. Recruitment. J. Biol. Chem..

[91] Palaniappan K.K., Bertozzi C.R. (2016). Chemical Glycoproteomics. Chem. Rev..

[92] Agard N.J., Bertozzi C.R. (2009). Chemical approaches to perturb, profile, and perceive glycans. Acc. Chem. Res..

[93] Richardson J.J., Ejima H. (2019). Surface Engineering of Extracellular Vesicles through Chemical and Biological Strategies. Chem. Mater..

[94] Lin S., Zhou S., Yuan T. (2020). The “sugar-coated bullets” of cancer: Tumor-derived exosome surface glycosylation from basic knowledge to applications. Clin. Transl. Med..

[95] Riley N.M., Bertozzi C.R., Pitteri S.J. (2020). A Pragmatic Guide to Enrichment Strategies for Mass Spectrometry-based Glycoproteomics. Mol. Cell. Proteom..

[96] Riley N.M., Malaker S.A., Driessen M.D., Bertozzi C.R. (2020). Optimal Dissociation Methods Differ for N- and O-Glycopeptides. J. Proteome Res..

[97] Marie A., Ray S., Lu S., Jones J., Ghiran I., Ivanov A.R. (2021). High-Sensitivity Glycan Profiling of Blood-Derived Immunoglobulin G, Plasma, and Extracellular Vesicle Isolates with Capillary Zone Electrophoresis-Mass Spectrometry. Anal. Chem..

[98] Stadlmann J., Taubenschmid-Stowers J., Wenzel D., Gattinger A., Dürnberger G., Dusberger F., Elling U., Mach L., Mechtler K., Penninger J.M. (2017). Comparative glycoproteomics of stem cells identifies new players in ricin toxicity. Nature.

[99] Fang P., Ji Y., Silbern I., Doebele C., Ninov M., Lenz C., Oellerich T., Pan K.-T., Urlaub H. (2020). A streamlined pipeline for multiplexed quantitative site-specific N-glycoproteomics. Nat. Commun..

[100] Ye Z., Mao Y., Clausen H., Vakhrushev S.Y. (2019). Glyco-DIA: A method for quantitative O-glycoproteomics with in silico-boosted glycopeptide libraries. Nat. Methods..

[101] Lu L., Riley N.M., Shortreed M.R., Bertozzi C.R., Smith L.M. (2020). O-Pair Search with MetaMorpheus for O-glycopeptide characterization. Nat. Methods.

[102] Polasky D.A., Yu F., Teo G.C., Nesvizhskii A.I. (2020). Fast and comprehensive N- and O-glycoproteomics analysis with MSFragger-Glyco. Nat. Methods.

[103] Royo F., Cossío U., de Angulo A.R., Llop J., Falcon-Perez J.M. (2019). Modification of the glycosylation of extracellular vesicles alters their biodistribution in mice. Nanoscale.

[104] Nishida-Aoki N., Tominaga N., Kosaka N., Ochiya T. (2020). Altered biodistribution of deglycosylated extracellular vesicles through enhanced cellular uptake. J. Extracell. Vesicles.

[105] Jaroentomeechai T., Stark J.C., Natarajan A., Glasscock C.J., Yates I.E., Hsu H.J., Mrksich M., Jewett M.C., DeLisa M. (2018). Single-pot glycoprotein biosynthesis using a cell-free transcription-translation system enriched with glycosylation machinery. Nat. Commun..

[106] Kightlinger W., Duncker K.E., Ramesh A., Thames A.H., Natarajan A., Stark J.C., Yang A., Lin L., Mrksich M., DeLisa M. (2019). A cell-free biosynthesis platform for modular construcion of protein glycosylation pathways. Nat. Commun..

[107] Moremen K.W., Haltiwanger R.S. (2019). Emerging structural insights into glycosyltransferase-mediated synthesis of glycans. Nat. Chem. Biol..

[108] Fernández-Tejada A., Brailsford J., Zhang Q., Shieh J.-H., Moore M.A.S., Danishefsky S.J. (2015). Total synthesis of glycosylated proteins. Top. Curr. Chem..

[109] Wen L., Edmunds G., Gibbons C., Zhang J., Gadi M.R., Zhu H., Fang J., Liu X., Kong Y., Wang P.G. (2018). Toward automation enzymatic synthesis of oligosaccharides. Chem. Rev..

[110] Chaffey P.K., Guan X., Li Y., Tan Z. (2018). Using chemical synthesis to study and apply protein glycosylatin. Biochemistry.

[111] Dusoswa S.A., Horrevorts S.K., Ambrosini M., Kalay H., Paauw N.J., Nieuwland R., Pegtel M.D., Würdinger T., Kooyk Y.V., Garcia-Vallejo J.J. (2019). Glycan modification of glioblastoma-derived extracellular vesicles enhances receptor-mediated targeting of dendritic cells. J. Extracell. Vesicles..

[112] Choi E.S., Song J., Kang Y.Y., Mok H. (2019). Mannose-modified serum exosomes for the elevated uptake to murine dendritic cells and lymphatic accumulation. Macromol. Biosci..

[113] Tan Z., Cao L., Wu Y., Wang B., Song Z., Yang J., Cheng L., Yang X., Zhou X., Dai Z. (2020). Bisecting GlcNAc modification diminishes the pro-metastatic functions of small extracellular vesicles from breast cancer cells. J. Extracell. Vesicles.

[114] Jafari D., Shajari S., Jafari R., Mardi N., Gomari H., Ganji F., Moghadam M.F., Samadikuchaksaraei A. (2020). Designer Exosomes: A New Platform for Biotechnology Therapeutics. BioDrugs.

[115] Chen W., Smeekens J.M., Wu R. (2015). Systematic and site-specific analysis of N-sialo-glycosylated proteins on the cell surface by integrating click chemistry and MS-based proteomics. Chem. Sci..

[116] Song S., Shim M.K., Lim S., Moon Y., Yang S., Kim J., Hong Y., Yoon H.Y., Kim I.-S., Hwang K.Y. (2020). In Situ One-Step Fluorescence Labeling Strategy of Exosomes via Bioorthogonal Click Chemistry for Real-Time Exosome Tracking In Vitro and In Vivo. Bioconjug. Chem..

[117] Kim E., Koo H. (2019). Biomedical applications of copper-free click chemistry: In vitro, in vivo, and ex vivo. Chem. Sci..

[118] Karver M.R., Weissleder R., Hilderbrand S.A. (2012). Bioorthogonal Reaction Pairs Enable Simultaneous, Selective, Multi-Target Imaging. Angew. Chem. Int. Ed. Engl..

[119] Smyth T., Petrova K., Payton N.M., Persaud I., Redzic J.S., Graner M.W., Smith-Jones P., Anchordoquy T.J. (2014). Surface Functionalization of Exosomes Using Click Chemistry. Bioconjug. Chem..

[120] Xu L., Faruqu F.N., Liam-Or R., Abed O.A., Li D., Venner K., Errington R., Summers H., Wang J.T.-W., Al-Jamal K.T. (2020). Design of experiment (DoE)-driven in vitro and in vivo uptake studies of exosomes for pancreatic cancer delivery enabled by copper-free click chemistry-based labelling. J. Extracell. Vesicles.

[121] Chiang C., Chen C. (2019). Toward characterizing extracellular vesicles at a single-particle level. J. Biomed. Sci..

[122] Wu D., Yan J., Shen X., Sun Y., Thulin M., Cai Y., Wik L., Shen Q., Oelrich J., Qian X. (2019). Profiling surface proteins on individual exosomes using a proximity barcoding assay. Nat. Commun..

[123] Ko J., Wang Y., Carlson J.C.T., Marquard A., Gungabeesoon J., Charest A., Weitz Z., Pitter M.J., Weissleder R. (2020). Single Extracellular Vesicle Protein Analysis Using Immuno-Droplet Digital Polymerase Chain Reaction Amplification. Adv. Biosyst..

[124] Kim D., Woo H., Lee C., Min Y., Kumar S., Sunkura V., Jo H.-G., Lee Y.J., Kim J., Ha H.K. (2020). EV-Ident: Identifying Tumor-Specific Extracellular Vesicles by Size Fractionation and Single-Vesicle Analysis. Anal. Chem..

[125] Zhang J., Shi J., Zhang H., Zhu Y., Liu W., Zhang K., Zhang Z. (2020). Localized fluorescent imaging of multiple proteins on individual extracellular vesicles using rolling circle amplification for cancer diagnosis. J. Extracell. Vesicles.

[126] Löf L., Ebai T., Dubois L., Wik L., Ronquist G. (2016). Detecting individual extracellular vesicles using a multicolor in situ proximity ligation assay with flow cytometric readout. Sci. Rep..

[127] Larssen P., Wik L., Czarnewski P., Eldh M., Löf L., Ronquist K.G., Dubois L., Freyhult E., Gallant C.J., Oelrich J. (2017). Tracing Cellular Origin of Human Exosomes Using Multiplex Proximity Extension Assays. Mol. Cell. Proteom..

[128] Han C., Kang H., Yi J., Kang M., Lee H., Kwon Y., Jung J., Lee J., Park J. (2021). Single-vesicle imaging and co-localization analysis for tetraspanin profiling of individual extracellular vesicles. J. Extracell. Vesicles.

[129] Lee K., Fraser K., Ghaddar B., Yang K., Kim E., Balaj L., Chiocca E.A., Breakefield X.O., Lee H., Weissleder R. (2018). Multiplexed Profiling of Single Extracellular Vesicles. ACS Nano.

[130] Gyuris A., Navarrete-Perea J., Jo A., Cristea S., Zhou S., Fraser K., Wei Z., Krichevsky A.M., Weissleder R., Lee H. (2019). Physical and Molecular Landscapes of Mouse Glioma Extracellular Vesicles Define Heterogeneity. Cell Rep..

[131] Fraser K., Jo A., Giedt J., Vinegoni C., Yang K.S., Peruzzi P., Chiocca E.A., Breakefield X.O., Lee H., Weissleder R. (2018). Characterization of single microvesicles in plasma from glioblastoma patients. Neuro Oncol..

[132] Zhang Y., Zhao W., Mao Y., Chen Y., Wang S., Zhong Y., Su T., Gong M., Du D., Lu X. (2020). Site-specific N-glycosylation Characterization of Recombinant SARS-CoV-2 Spike Proteins. Mol. Cell. Proteom..

[133] Richmond P., Hatchuel L., Dong M., Ma B., Hu B., Smolenov I., Li P., Liang P., Han H.H., Liang J. (2021). Safety and immunogenicity of S-Trimer (SCB-2019), a protein subunit vaccine candidate for COVID-19 in healthy adults: A phase 1, randomised, double-blind, placebo-controlled trial. Lancet.

[134] Taylor N.P. Drug Delivery Capricor Touts Exosomes as Better mRNA Delivery Vehicle. www.fiercepharma.com/drug-delivery/capricor-touts-exosomes-as-better-mrna-delivery-vehicle.

[135] Chernykh A., Kawahara R., Thaysen-Andersen M. (2021). Towards structure-focused glycol-proteomics. Biochem. Soc. Trans..

[136] Sobotzki N., Schafroth M.A., Rudnicka A., Koetemann A., Marty F., Goetze S., Yamauchi Y., Carreira E.M., Wollscheid B. (2018). HATRIC-based identification of receptors for orphan ligands. Nat. Commun..

[137] Frei A.P., Jeon O.-Y., Kilcher S., Moest H., Henning L.M., Jost C., Plückthun A., Mercer J., Aebersold R., Carreira E.M. (2012). Direct identification of ligand-receptor interactions on living cells and tissues. Nat. Biotechnol..

[138] Frei A.P., Moest H., Novy K., Wollscheid B. (2013). Ligand-based receptor identification on living cells and tissues using TRICEPS. Nat. Protoc..

[139] Tremblay T.L., Hill J.J. (2017). Biotin-transfer from a trifunctional crosslinker for identification of cell surface receptors of soluble protein ligands. Sci. Rep..

[140] Miyajima R., Sakai K., Otani Y., Wadatsu T., Sakata Y., Nishikawa Y., Tanaka M., Yamashita Y., Hayashi M., Kondo K. (2020). Novel Tetrafunctional Probes Identify Target Receptors and Binding Sites of Small-Molecule Drugs from Living Systems. ACS Chem. Biol..

[141] Yang Y., Verhelst S.H.L. (2013). Cleavable trifunctional biotin reagents for protein labelling, capture and release. Chem. Commun..

[142] Flynn R.A., Pedram K., Malaker S.A., Batista P.J., Smith B.A.H., Johnson A.G., George B.M., Majzoub K., Villalta P.W., Carette J.E. (2021). Small RNAs are modified with N-glycans and displayed on the surface of living cells. Cell.

[143] Wei Z., Batagov A.O., Schinelli S., Wang J., Wang Y., EL Fatimy R., Rabinovsky R., Balaj L., Chen C.C., Hochberg F. (2017). Coding and noncoding landscape of extracellular RNA released by human glioma stem cells. Nat. Commun..

[144] Parker C.G., Pratt M.R. (2020). Click Chemistry in Proteomic Investigations. Cell.

